# Inflammaging as a prodrome to Alzheimer's disease

**DOI:** 10.1186/1742-2094-5-51

**Published:** 2008-11-11

**Authors:** Brian Giunta, Francisco Fernandez, William V Nikolic, Demian Obregon, Elona Rrapo, Terrence Town, Jun Tan

**Affiliations:** 1 Neuroimmunology Laboratory, Department of Psychiatry, Behavioral Medicine, Institute for Research in Psychiatry, University of South Florida, College of Medicine, Tampa, FL 33613, USA; 2 Rashid Laboratory for Developmental Neurobiology Department of Neurosurgery, University of South Florida, College of Medicine, Tampa, Florida 33613, USA; 3 Department of Neurosurgery, Maxine Dunitz Neurosurgical Institute, Cedars-Sinai Medical Center, Los Angeles, CA 90048, USA; 4 Department of Biomedical Sciences, Cedars-Sinai Medical Center, Los Angeles, CA 90048, USA; 5 Department of Medicine, David Geffen School of Medicine, University of California, Los Angeles, Los Angeles, CA 90048, USA

## Abstract

Recently, the term "inflammaging" was coined by Franceshci and colleagues to characterize a widely accepted paradigm that ageing is accompanied by a low-grade chronic up-regulation of certain pro-inflammatory responses. Inflammaging differs significantly from the traditional five cardinal features of acute inflammation in that it is characterized by a relative decline in adaptive immunity and T-helper 2 responses and is associated with increased innate immunity by cells of the mononuclear phagocyte lineage. While the over-active innate immunity characteristic of inflammaging may remain subclinical in many elderly individuals, a portion of individuals (postulated to have a "high responder inflammatory genotype") may shift from a state of "normal" or "subclinical" inflammaging to one or more of a number of age-associated diseases. We and others have found that IFN-γ and other pro-inflammatory cytokines interact with processing and production of Aβ peptide, the pathological hallmark feature of Alzheimer's disease (AD), suggesting that inflammaging may be a "prodrome" to AD. Although conditions of enhanced innate immune response with overproduction of pro-inflammatory proteins are associated with both healthy aging and AD, it is suggested that those who age "well" demonstrate anti-inflammaging mechanisms and biomarkers that likely counteract the adverse immune response of inflammaging. Thus, opposing the features of inflammaging may prevent or treat the symptoms of AD. In this review, we fully characterize the aging immune system. In addition, we explain how three novel treatments, (*1*) human umbilical cord blood cells (HUCBC), (*2*) flavanoids, and (*3*) Aβ vaccination oppose the forces of inflammaging and AD-like pathology in various mouse models.

## Introduction

Some two thousand years ago, Celsus first described 4 cardinal signs of inflammation: (*1*) redness, (*2*) swelling, (*3*) heat, and (*4*) pain. Shortly thereafter a fifth sign, loss of function, was added by Galen. Recently, the term "inflammaging" was coined by Franceshci and colleagues to characterize a widely accepted paradigm that ageing is accompanied by a low-grade chronic up-regulation of certain inflammatory responses [1-8]. Inflammaging differs significantly from the traditional five cardinal features of acute inflammation in that it is a (*a*) low-grade, (*b*) controlled, (*c*) asymptomatic, (*d*) chronic, and (*e*) systemic state of inflammation [9]. This systemic inflammatory response is evidenced by increased serum levels of pro-inflammatory cytokines (IL-6, IL-15, IL-8) [10-15] and other inflammatory biomarkers, such as coagulation factors [16-18]. Additionally, subclinical infection with common viruses such as cytomegalovirus (CMV) is a hallmark feature of inflammaging. There also may be a genetic component, as suggested by studies on alleles coding for mediators of inflammation including cytokines and coagulation factors [19-21]. Finally, reactive oxygen species (ROS) cause amplification of cytokine release, fueling a self-perpetuating positive feedback loop. The end result of this cycle is a chronic and systemic pro-inflammatory state where both tissue damaging and healing mechanisms operate simultaneously. Over decades, the opposing forces are likely critical perpetrators of ageing and age related disease, leading to an accumulation of subtle tissue damage [3,5-8].

## Inflammaging is characterized by a relative decline in adaptive immunity and T-helper 2 responses and is associated with increased cell mediated responses

According to Franceschi's original description of inflammaging, innate immunity progresses to a chronically active state secondary to exhaustion of the more evolutionary recent adaptive (specific) immune system [22]. This exhaustion is in large part due to age-associated reduction of T-cells for various reasons including thymic involution [23-25], as well as fewer bone marrow early progenitor B cells [26]. In early life, naïve T cells are activated by contact with antigens. They then differentiate into effector or memory cells. Because the quantity of T-cells in healthy individuals is stable over the adult lifespan, peripheral T cell turnover of pre-existing populations in the thymus is required for replacement of T cells in relatively young individuals [27-30].

Together with the diminution of adaptive immunity that occurs in inflammaging, there is also an increase in the number of antigen-experienced cells and a decrease in the number of naïve T cells in the circulation, which results in accumulation of incompetent memory lymphocytes [31]. These cells likely clonally expanded and became effector memory T-cells that were competent at one time, but then lost their antigen-specific function due to their age. This phenomenon is believed to be owed to life-long antigenic stress from immunosurveillance against persistent viruses, especially CMV [32]. This accumulation of a limited repertoire of "megaclones" of effector memory CD8^+ ^T cells results from long-term, chronic exposure to antigens over time frames much longer than during evolution of the human immune system [28,33-39]. The net result of this is (*1*) to reduce adaptive immunity to previously encountered pathogens, and (*2*) to weaken the host adaptive immune response to novel pathogens due to a reduction in the diversity of the antigen-recognition repertoire with age. In fact, based on analysis of human T-cell receptor (TCR) Vβ chain usage, the antigen-recognition repertoire decreases from approximately 10^8 ^in young adults to 10^6 ^in the elderly [40]. Moreover, CD8^+ ^T-cells in the elderly display significantly decreased ability to secrete interferon-gamma (IFN-γ) when stimulated by cognate antigen in comparison to younger age groups [36,38]. Also, naïve CD4+ T-cells from old humans and mice show decreased responsiveness to TCR stimulation and altered profiles of cytokine production versus naïve CD4^+ ^T-cells from young hosts. Likewise, the helper function of naïve CD4^+ ^T-cells for antibody production by B cells is also decreased [41]. The decline in responsiveness of naïve CD4^+ ^T cells is due to the chronologic age of the cells themselves, and not of the individual host [41], suggesting that long-term maintenance of naïve CD4^+ ^T-cells through homeostatic cytokines may not have a positive impact on their function. Indeed, naïve CD4+ T-cells that have undergone homeostatic cell divisions proliferate less and secrete less IL-2 in response to antigen than do naïve CD4+ T-cells that have not undergone homeostatic division [41].

Unlike naïve lymphocytes, memory CD4^+ ^T lymphocytes are long-lived and relatively competent with age. These antigen-experienced T-cells, when isolated from healthy elderly humans and healthy old mice, respond normally to antigen-induced proliferation *in vitro *[42] and those generated at a young age respond well to antigens over time. Conversely, naïve CD4^+ ^T-cells derived in old age respond poorly [43]. Together, these studies point to an age-associated defect in memory CD4+ T cells which may originate from defects of aged naïve CD4^+ ^T-cells that have reduced clonal diversity and proliferation potential. Interestingly, changes of the ratio of memory to effector CD4^+ ^T-cell subsets with age have also been implicated in deficient adaptive immune responses to viral infections and vaccines [44].

An additional important feature of inflammaging which contributes to defective T-cell function and adaptive immunity is accumulation of CD28^-^CD8^+ ^T-cells and the loss of naïve CD8+ T-cells. These T-cells are absent in newborns while composing some 85% of circulating CD8+ T-cells in the elderly. The accumulation of CD28^-^CD8^+ ^T-cells was also shown in patients with viral infections such as CMV [45]. CD28^-^CD8^+ ^T-cells may signify terminal differentiation from the CD28+CD8^+ ^subset after repeated antigenic stimulation. Functionally, CD28^-^CD8^+ ^T-cells have a reduced proliferative response to TCR stimulation but exhibit normal or even enhanced cytotoxic capacity and are resistant to apoptosis [46]. Furthermore, several studies have demonstrated the virtual disappearance of naïve CD8^+ ^T-cells in the elderly associated with a significant increase in the proportions of differentiated effector memory and effector CD8^+ ^T-cells in comparison to younger individuals. Interestingly, most of these cells in the elderly do not have short telomeres. Taken together, these data suggest that, over the course of life, these T-cell populations have undergone a process of end-stage differentiation and that persistent infection with common pathogens, such as CMV, induces chronic stimulation of specific T cells that leads to terminal differentiation to senescent cells with an altered functional capability [30,47-50]. Further, it seems that clonal expansion of CD28^-^CD8^+^T-cells seems directly responsible for increased infection rates and the common failed response to vaccines in the elderly [51].

Decreased adaptive immunity characteristic of inflammaging also involves changes in the B-cell repertoire not unlike those observed in the T-cell pool [52-54,54]. The quality of the humoral immune response is decreased with age owing to low serum immunoglobulin concentrations and low numbers of antigen-specific, immunoglobulin-secreting plasma cells. Further, antibody specificity, isotype and affinity changes are typical features of old age [54]. For example, immunoglobulins produced in aged mice have lower affinity and are less protective versus those of young animals [55]. This may be secondary to aging's adverse effects on the germinal centre reaction in secondary lymphoid tissues [56], and leads to a diminished quantity of germinal centres in response to tetanus toxoid stimulation [57]. The quantity of B cells also decreases in the elderly (58). At the cellular level, alteration in immunoglobulin generation (through class switching) in B cells is observed in aged individuals [59], which may contribute to the decline of the adaptive immune response in the elderly. Also, age is positively associated with B cell malignancies in older adults with oligoclonally expanded B cells [30].

The weakened adaptive immune responses characteristic of inflammaging may also be caused by deficient MHC class II antigen presentation by dendritic cells (DCs) and macrophages, or in the case of the brain, microglia [60,61]. Although these cells are part of the innate immune system, they are critical for initiation of adaptive immunity. That is, activation of these phagocytic cells *via *pattern recognition receptors (PRRs), such as toll-like receptors (TLRs), mediates their maturation and migration to secondary lymphoid organs where they present antigens to T-cells [62,63]. Studies have indicated that this diminished MHC class II expression over the life span is typical in both humans and mice [64,65]. Not only do innate immune cells down-regulate MHC II, but decreased numbers of Langerhans cells (LCs; the DCs of the skin) have been observed consistently in both elderly humans and old mice. LC migration to lymph nodes is also decreased in old mice; possibly secondary to reduced availability of IL-1β, a cytokine which provides autocrine stimulus for migration and induces TNF-α production by adjacent keratinocytes, which also promotes LC mobilization [66]. Additionally, DCs from elderly people may under-express costimulatory receptors and IL-12, thus impairing their ability to induce effector T-cell differentiation. In addition, the production of IL-10 is elevated in elderly individuals and may inhibit DC maturation and macrophage function [60]. Further, it was also demonstrated that IFN-γ-mediated induction of the MHC II gene (*H2-Ab1*) is impaired in macrophages from aged mice due to the decreased binding of transcription factors to the promoter [64]. This lack of proper activity of the innate arm of immunity has also been associated with upregulated pro-inflammatory responsiveness. That is, activated macrophages from aged humans and mice produce higher amounts of the proinflammatory paracrine molecule prostaglandin E2 than younger individuals, which inhibits surface expression of MHC class II [65]. In addition, we also found proinflammatory activation of mouse microglia inhibits their ability to phagocytose Alzheimer's amyloid-beta (Aβ) peptide [67].

Coincident with this phenomenon, either due to a "release" of adaptive control on innate immunity and/or a compensatory mechanism for lack of effectiveness of adaptive immunity, is over-activation of certain pro-inflammatory responses [4,7,22] by mononuclear phagocytes (cells of the innate immune system) [68]. These cells are the first to arrive at sites of infection or tissue damage. Their physiologic role is to initiate an inflammatory response, phagocytose the pathogen or foreign body, recruit natural killer (NK) cells, and facilitate the terminal differentiation and migration of DCs that will modulate the T cell-mediated adaptive immune response. [32]. These cells, including neutrophils and macrophages, activate innate immunity *via *their PRRs [69] which induce a pro-inflammatory response *via *downstream nuclear factor-kappa beta (NF-kβ) and mitogen associated protein kinase (MAPK) signaling [70,71]. Indeed, studies in both elderly humans and old mice substantiate the notion that inflammaging constitutes a gradual loss of adaptive and increased activity of mononuclear cell innate immunity demonstrated by pro-inflammatory cell responses to mitogens [30,32]. For example, peripheral blood monocytes from elderly individuals generated higher amounts of tumor necrosis factor alpha (TNF-α) and interleukin 1 beta (IL-1β) in comparison to cells from young subjects [72]. Furthermore, the data regarding cytokine and chemokine secretion in several studies using human monocytes from aged or young individuals activated *in vitro *with mitogens or the bacterial endotoxin lipopolysacharride (LPS), for the most part, demonstrate an increase in the levels of pro-inflammatory factors such as IL-6 and IL-8 [73-79]. As noted above, activated macrophages from aged mice and humans produce more prostaglandin E2 than younger individuals, and this proinflammatory coagulation factor suppresses IL-12 secretion, decreases APC surface expression of MHC II, and enhances IL-10 secretion which inhibits T-cell function. This increased prostaglandin E2 is due to enhanced expression of inducible cyclooxygenase [80]. We suggest that, underlying this increased proinflammatory signaling by mononuclear cells of the innate immune system is the previously mentioned increase in the number of antigen-experienced T cells and the decrease in the number of naïve cells in the elderly. The antigen-experienced T cells (observed as large oligoclonal expansions which dominate the repertoire) have lost the important costimulatory molecule CD28 and have a polarized pro-inflammatory T-helper 1 (Th1) profile [81]. Cytokines derived from Th1 cells are associated with cell mediated pro-inflammatory responses, while Th2 cytokines act on B cells to enhance adaptive immune responses (including antibody production) by promoting activation and differentiation. Importantly, an imbalanced Th1/Th2 ratio can change the cytokine microenvironment in lymphatic tissues and trigger the chronic pro-inflammatory innate immune processes characteristic of inflammaging [22]. It is important to note that there are conflicting reports on the basic notion that an imbalance in Th1/Th2 immunity is best characterized in the elderly by overactive Th1 and underactive Th2 cytokine production. For instance, the production of the Th1 cytokine IFN- has been reported to be high [82,83] or low [84-87] during aging. Such discrepancies may result from variations among species, strains, organs, culture systems, or inter-individual differences during the course of the aging process. To circumvent such confounds, studies focusing on provoking an antigen-specific immune response, for example by vaccination, have proved powerful. For example, influenza vaccination induces protective antibodies in only 40–70% of the elderly [88,89]. Saurwein-Teissl and colleagues (2002) analyzed a very homogenous "model" cohort of elderly persons, none of whom produced specific antibodies one month after influenza vaccination. Using this homogenous cohort, the lack of antibody production following immunization was positively associated with the increased occurrence of the expanded CD8^+^CD28^- ^T cell subset. These clones, which produce large amounts of IFN-γ, are strongly suggested to be the basis for a change in the polarization of the immune system toward a Th1 profile in elderly persons and the development of age-related immune deficiency [90]. Moreover, we would suggest that the response to a vaccine may be used as an assessment of inflammaging, and thus risk for age-related disease.

As further detailed below, proinflammatory cytokines associated with reduced adaptive and upregulated innate mononuclear cells (an integral feature of inflammaging) are now believed to exacerbate the pathology and disease course of age-related disorders including Alzheimer's disease (AD) [91-93]. For example, IFN-γ in combination with TNF-α triggers the production of Aβ peptide in human neuronal and extraneuronal cells [3] and increases the production of ROS by microglia. TNF-α can also further exacerbate Aβ toxicity [4].

## Does inflammaging perpetrate and/or exacerbate AD?

While the over-active innate immunity characteristic of inflammaging may remain sub-clinical in many elderly individuals for the reasons stated above, a portion of individuals (postulated to have a "high responder inflammatory genotype") may shift from a state of "normal" or "subclinical" inflammaging to one or more of any age-associated diseases. Simultaneously, aging is associated with an increase in production of oxygen-derived radicals, i.e., reactive oxygen species (ROS), with a concurrent decrease in the ability to defend against ROS. Indeed, many works have established oxidative stress and damage not only in the lesions of AD but also in neurons at risk of death [94-100].

It is interesting to note that Alois Alzheimer himself first noted what he termed "gliose" (English translation: *gliosis*, or inflammation of glia) when characterizing brain pathology in 1907 in the first AD case. Indeed microglia are the common source of pro-inflammatory and oxidative stress in AD as shown by consistent gross observations of upregulated inflammatory responses in brain (including products of activated microglia and astrocytes) regions that exhibit high levels of AD pathology such as the frontal and limbic cortices [101-103]. Conversely, these markers of inflammation or oxidative stress are absent or minimal in brain regions which are typically less susceptible to AD pathology, such as cerebellum. Upon analysis, inflammatory mediators and signs of oxidative stress are most robustly observed in regions of Aβ deposits and neurofibrillary tangles (NFT), classical AD hallmarks often associated with neurodegeneration [104]. For example, significant increases of an oxidized nucleoside derived from RNA, 8-hydroxyguanosine (8OHG), and an oxidized amino acid, nitrotyrosine, have been demonstrated in vulnerable neurons of AD patients [105]. Importantly, it was shown that oxidative damage is quantitatively greatest early in the disease and reduces with disease progression. Interestingly however, increases in Aβ deposition were associated with decreased oxidative damage [105]. Moreover, neurons with NFT show a 40%–56% decrease in relative 8OHG levels compared with neurons free of NFT. We would suggest that inflammaging involves increased oxidative damage prior to a diagnosis of AD (or at least early in AD [105,208] due to over-activation of certain pro-inflammatory responses [4,7,22] by mononuclear phagocytes (cells of the innate immune system) [68]. As AD worsens, amyloid deposition increases simultaneously with compensatory changes that reduce damage from ROS, which could include disregulation of T-cell responses [106]. This is further supported by findings in Tg2576 AD transgenic mice in which oxidative stress precedes Aβ deposition [107]. Also supporting this notion, patients with MCI or very mild AD show increased levels of lipid peroxidation and nucleic acid oxidation in postmortem brain, CSF, plasma, urine and peripheral leukocytes in conjunction with decreased levels of plasma antioxidants and total plasma antioxidant capacity [108-110].

Perhaps the most important piece of evidence to substantiate the pathogenic importance of inflammaging in AD comes from patients without history of dementia but who nonetheless exhibit sufficient Aβ/NFT at autopsy to otherwise qualify an AD diagnosis (so-called "high pathology controls") [111]. Interestingly, these patients demonstrate only modest elevations of proinflammatory markers compared with typical non-demented patients but dramatically less than AD patients [112]. Furthermore, there is a plethora of evidence indicating direct inflammatory toxicity in AD brain. For example, assembly of complement components ultimately leading to membrane attack complex formation and lysis of neuronal cells has been shown ultrastructurally [113]. In seeking to elaborate and extend upon the original theories postulated by Franceshci, it appears that inflammaging may serve as a prodrome or an exacerbating factor for development of AD [9,22]. Understanding AD in this context may shed new light on immunological treatment targets for AD, which may also apply to other neurodegenerative diseases including HIV associated dementia (HAD), Parkinson's Disease, and Multiple Sclerosis (MS).

Conditions of enhanced innate immune response with overproduction of pro-inflammatory proteins are associated with both healthy aging and AD [114-116]. The difference between these groups seems to be that those who age "well" demonstrate anti-inflammaging mechanisms and biomarkers that likely counteract the adverse immunity characteristic of inflammaging. For example, it seems that centenarians demonstrate anti-inflammatory responses which may delay or even stop emergence of many age-related diseases [9,117,118]. Some examples in centenarians are as follows: reduction of pro-inflammatory, pro-atherosclerotic properties of platelets [119], increased resistance to inhibitory actions of homocysteine on nitric oxide generation [119], low plasma membrane lipid peroxide levels, high membrane fluidity with normal content of sialic acid [120-123] and increased plasma cortisol, one of the most potent anti-inflammatory molecules [124,106]. Further, an example of a genetic anti-inflammaging mechanism in centenarians is increased frequency in males of the IL-10-1082GG genotype, which is associated with high production of this important anti-inflammatory cytokine [125]. Conversely, the same genotype is much less frequent in patients affected by AD [126]. Thus, it is believed that individuals who carry such "high responder" genotypes may suffer from diseases in which inflammaging could be a key eitiological disease component. This notion is supported by many studies indicating an increase of inflammatory parameters, such as complement factors, proinflammatory cytokines, and activation of microglia co-localized with amyloid plaques in the AD brain [127].

Others have also reported what would seem to be biomarkers of inflammaging in AD. One of these markers is neopterin, a blood compound produced by monocyte-derived macrophages upon stimulation with IFN-γ. Although it is virtually impossible to determine whether activation of the innate or acquired arms of the immune system is primary based on measurements of neopterin, in contrast to the well known marker of systemic immune activation, c-reactive protein (CRP), neopterin seems to be more associated with cell-mediated responses and is commonly used as a biomarker for this purpose [128,129]. Three studies to date have shown elevated levels of neopterin in AD patients compared to age-matched controls [130-132]. The authors suggest this elevated neopterin may be a vestigial result of serum immunity to CMV. That is, in the AD patients, 70% were positive for CMV antibody and CMV antibody concentrations correlated with concentrations of neopterin and CRP. As previously mentioned, CMV infection seems to be a critical factor in the diminution of adaptive immunity in the elderly as it confers expansion of a limited repertoire of CD8^+ ^T cells, which afterward lack full functionality [45,90]. A similar response to influenza vaccination in the elderly was described [90]. Thus, it has been proposed that enhanced cell-mediated innate immunity reflected by neopterin production in AD patients might be secondary to senescence of CMV-expanded T-cell clones [114]. It is of course difficult to determine from these clinical studies whether such factors as neopterin, CRP, and humoral responses are etiologically involved in AD, but it certainly appears that there is a positive correlation between these factors and AD.

Several studies have examined T-cell populations and activation markers in AD in peripheral blood (for a review see 188). Although total percentage of T-cells was not found to be altered [133,134], alterations of their function, differentiation and subset distribution have been demonstrated. Abnormal functioning of suppressor as well as Th cells has been described in AD patients [135,136]. Significantly decreased activation response of this adaptive immune cell in AD compared to age-matched controls has also been observed. The proliferative response of T-cells in AD seems reduced; however, this was not universally observed. While two studies found no alterations in the proliferative response of AD patient T-cells [137,138], four studies report a decrease in proliferation of AD patient T-cells [139-141]. Most recently, one study [109] found a significant decrease of CD3+ T lymphocytes and CD19+ B lymphocytes in AD patients. A slight increase of the CD4^+ ^and a decrease of the CD8^+ ^subpopulation was also observed, but this was not accompanied by a significant change in the CD4^+^/CD8^+ ^ratio, and CD16^+^CD56^+ ^cells were not altered. Schindowski and colleagues found increased apoptosis in CD4^+ ^T cells as well as NK cells in AD patients, while in aged individuals, all subsets were affected. This may be explained by the levels of Bcl-2 in T-cells, which were significantly increased in patients with mild AD. Apoptosis and Bcl-2 levels were also elevated in the APP^751SL ^× PS1^M146L ^transgenic mouse model of AD [142]. Together, these finding suggest a diminution of adaptive immunity reflected by dysregulated and altered T- cell distributions in AD [127]. However, as mentioned above, it is difficult to conclude from these clinical studies whether such alterations in the leukocyte repertoire of AD patients are etiologically involved in disease progression or simply epiphenomena. For this reason, future studies in mouse models of AD deficient in key innate/adaptive immune/inflammatory molecules will be important.

*In vitro *and *in vivo *studies by us and other groups suggest that IFN-γ and other pro-inflammatory cytokines interact with processing and production of the Aβ peptide [143,144]. For example, IFN-γ is a strong stimulator of Aβ_1–40 _and Aβ_1–42 _production *in vitro *in neurons and in other brain cells [143,144]. Even if IFN-γ produced in the periphery does not directly affect brain Aβ levels, increased peripheral production of Aβ at sites where IFN-γ levels are elevated might influence Aβ levels in the brain [145,146]. Thus, we and others have proposed adverse effects of altered immunity (upregulated innate mononuclear cell immunity/Th1 cytokine secretion and downregulated adaptive immunity) in AD patients, and this has led us and others to focus on three diverse treatment modalities in AD transgenic mice models which seem to "re-balance" pro- versus anti-inflammatory responses in the AD brain. These therapies are: *(1) *non-steroidal anti-inflammatory drugs (NSAIDs), (*2*) Aβ immunization, *(3) *human umbilical cord blood cells (HUCBC), and (*4*) plant-derived compounds known as flavonoids.

Finally,, we suggest that that inflammaging as a prodrome to AD fits well with the "2-hit hypothesis" of AD put forth by Zhu and colleagues stating that both oxidative stress and mitogenic dysregulation are necessary and sufficient to cause AD [147]. The first "hit" required for AD development is suggested to be the above discussed oxidative stress during MCI and early in the course of AD. This goes hand in hand with upregulation of innate immunity as part of inflammaging. The second "hit" is also suggested to occur during this same prodromal time period during inflammaging and disregulation of neuronal mitotic proteins [148,149]. Indeed, cell cycle markers occur prior to the appearance of gross cytopathological changes in AD [150]. In further support of this idea, mitotic events have been shown to occur in pre-AD patients with MCI which represents a time point where inflammaging effects are near their maximum, ie during the prodromal stage of AD [151].

## Re-balancing innate and adaptive immunity as a treatment paradigm for AD NSAIDs and Aβ immunization: from mice to humans

Several observational studies [152-154] have shown that use of various anti-inflammatory agents is associated with reduced risk of developing AD. After preliminary studies produced encouraging results with nonsteroidal anti-inflammatory drugs (NSAIDs) for AD treatment, [155,156] a series of larger studies were launched that tested glucocorticoid therapy, [157] NSAIDs, [158-160] and hydroxychloroquine [161] on patients with AD and MCI and also cognitively normal individuals at risk for AD. The results of these trials to date have been negative. None of the NSAIDs tested in early trials had amyloid-reducing activity. Newer NSAIDS including LY450139 [162,163] and R-flurbiprofen [164] do have this activity. LY450139, given in doses ranging from 5–50 mg/day over 14 days to normal human volunteers, decreased plasma Aβ concentrations up to 40% in a dose-dependent manner. Unfortunately however, CSF Aβ concentrations were unchanged. Furthermore, at the 50 mg/d dose, adverse events that were possibly drug-related were noted. R-flurbiprofen is currently in phase III testing (*N *= 2400). In a one-year phase II study (*N *= 207) of subjects with mild-to-moderate AD receiving 400 mg b.i.d., 800 mg b.i.d., or placebo, statistical significance was not reached in any memory measures. However, a subset of mild patients receiving the 800 mg b.i.d dose (who developed high blood levels of the drug) demonstrated significant benefits in activities of daily living and overall cognitive function [164]. A recent trial of the new NSAID, trifusal, which also has γ-secretase inhibition activity, was also negative [165]. The primary outcome measure was the change in score on a cognitive assessment (an expanded version of the ADAScog). Of course, these above-named negative trials do not offer evidence that a different NSAID, another dose, a different duration, or another study population would not yield positive results. We and others have pre-clinically examined three diverse therapies; all which lower cerebral amyloid deposits: (*1*) Aβ immunization, (*2*) human umbilical cord blood cell (HUBC) transplantation, and (*3*) flavonoid supplementation, which we suggest may show promise as combination or single treatment(s) in AD patients.

Immunization against Aβ peptide was first explored by Schenk and colleagues at Elan Pharmaceuticals. The vaccine was prepared from Aβ_1–42 _peptide (the presumed pathogenic form of Aβ found in AD plaques) and complete Freund's adjuvant. This original Aβ vaccine conferred both therapeutic and prophylactic value in terms of reducing CNS amyloid deposits in a transgenic mouse model that overexpressed mutant human APP. In addition to augmenting adaptive immunity, the Elan vaccine generated high-titre Aβ-specific antibodies [166]. The following year, another member of the team at Elan Pharmaceuticals reported a different version of the vaccine using passive transfer of Aβ-specific antibodies to transgenic AD mice [167]. This version of the vaccine was also efficacious, although less-so than the original "active" Aβ vaccine developed by Schenk and co-workers. Elan/Wyeth then developed the vaccine for clinical trials in 2001 (AN-1792), but approximately 6% of patients developed aseptic meningoencephalitis, which was most likely attributed to brain infiltration of activated Aβ-specific T-cells. Two years later, consent for long-term clinical follow-up, post-mortem neuropathological examination, or both, was sought from 80 patients (or their caretakers) who had entered the original Elan AN-1792 phase I trial. The follow-up study was completed in 2006. Twenty participants-15 in the AN1792 group, 5 in the placebo group-died before follow-up started. A further 22 patients-19 in the AN1792 group, three in the placebo group-died during follow-up. Nine of the deceased subjects, all in the AN1792 group, had given consent for post-mortem analysis; one of these who did not die with AD was excluded [168]. In the remaining eight patients who received immunization and who were examined neuropathologically, mean Aβ load was lower than in an unimmunised control group matched for age at death (2.1% [SE 0.7] in treated participants vs 5.1% [0.9] in controls; mean difference 3.0%, 95% CI 0.6–5.4; p = 0.02). Although there was variation in load and degree of plaque removal among immunized subjects, the degree of plaque removal varied significantly with mean antibody response attained during the treatment study period (Kruskal-Wallis p = 0.02). Seven of the eight immunised patients who underwent post-mortem assessment, including those with essentially complete plaque removal, still had severe end stage dementia before death. Further, in the whole cohort, there was no evidence of improved survival (hazard ratio 0.93, 95% CI 0.43–3.11; p = 0.86) or of an extension of the time until severe dementia (1.18, 0.45–3.11; p = 0.73) in the AN1792 group versus the placebo group. Thus, it seems that immunization lowered amyloid load but not progressive neurodegeneration [168].

There have been a number of other attempts at Aβ vaccination [169-172]. However, it should be noted that none of these other Aβ vaccines have yet to be tested in humans, and none of the Aβ vaccines tested in mice (with the exception of one unconfirmed report) have produced the meningoencephalitis that occurred in a subset of vaccinated AD patients [173]. Despite this limitation of the mouse models, we have developed a transdermal method to effectively deliver an Aβ vaccine. This version of the vaccine decreases cerebral amyloidosis and does not promote infiltration of T-cells into the brain; but, again, the latter data should be interpreted in the context of lack of brain T-cell infiltrates in most AD mouse models receiving the Aβ vaccine. Specifically, we designed and tested a novel transcutaneous (t.c) Aβ_1–42 _vaccination strategy along with cholera toxin (CT) as the adjuvant. The vaccine was tested on wild-type mice as well as on PSAPP and Tg2657 transgenic mouse models of AD, which over-express mutant human APP and develop brain β-amyloid plaques. Additionally, the Tg2657 model develops AD-like cerebral amyloid angiopathy (CAA); a pathology which occurs in the majority of AD patients. We found that the vaccine impacted both innate and adaptive immunity in both wild-type and AD transgenic mice and mitigated AD pathology in the transgenic mice [174].

The t.c. Aβ vaccine stimulated an innate immune response in the non-transgenic mice as it caused LCs to migrate to dermal layers containing the Aβ peptide. This response was less frequently observed with administrations of CT only or with PBS, suggesting that this effect may mediate the initial immune response to the vaccine. In addition, there is evidence from our experiments that the immunization stimulated an adaptive immune response as there was an increase in Aβ antibody concentrations (particularly of the IgG1 subtype) as well as Aβ-specific immune responses in splenocytes from vaccinated mice. Since IgG1 antibodies were produced at higher levels than IgG2a or IgG2b, and since there was an 8-fold increase in the secretion of the cardinal Th2 cytokine IL-4 from Aβ "recall" challenged splenocytes, it seems that the t.c. Aβ vaccine promotes an anti-inflammatory Th2 adaptive immune response, which appears similar to the immune responses observed by our group when employing an i.p. Aβ vaccine protocol similar to the original Schenk and colleagues report [175,176]. The AD mouse models produced a similar adaptive immune response after t.c. Aβ vaccination, with increased concentrations of Aβ antibody and Aβ "recall" challenge-induced production of cytokines by splenocytes.

The above-mentioned alterations in adaptive immunity were associated with reduction of cerebral β-amyloid deposits by 4G8 immunohistochemistry and Congo red histochemistry, and levels of insoluble Aβ_1–40,42 _were reduced by 50% and 54%, respectively. In addition, Aβ deposits were reduced by 42–58% across hippocampal and cortical areas, brain regions classically regarded as most sensitive to AD-type pathological changes. It deserves mentioning that we did not observe the negative side-effects of cerebral T-cell infiltration (as detected by CD3 antibody) or cerebral microhemorrhage; but, as mentioned above for the former, most Aβ vaccines do not produce this effect in mice. The lack of microhemorrhage in the brains of mice receiving the t.c. Aβ vaccine suggests that the Aβ antibody response was not pathogenic, as the vaccine did not promote break-down of the blood-brain barrier. We also observed an inverse correlation between plasma and brain-soluble Aβ, signifying that circulating Aβ antibodies may be important for clearing Aβ from the brain to the blood; consistent with the "peripheral sink hypothesis" of DeMattos and colleagues [177].

## Modulating immunity in AD with human umbilical cord blood cells

Just as vaccination strategies modulate immunity in AD by boosting adaptive immunity, human umbilical cord blood cells (HUCBC) have been shown to have immunomodulatory properties by opposing pro-inflammatory Th1 and stimulating anti-inflammatory Th2 responses in animal models of stroke and AD [178,179]. HUCBCs have also shown therapeutic benefit in other neuroinflammatory conditions including multiple sclerosis, amyotrophic lateral sclerosis, neurodegenerative macular degeneration, and Parkinson's disease [180-182]. Our recent study showed that intravenous HUCBC infusion into transgenic AD mice resulted in diminished cerebral Aβ/β-amyloid pathology and down-regulation of pro-inflammatory responses in the brain and in the periphery [183]. We attempted to localize HUCBCs to the brain, but were unable to find CNS penetration of these cells, leading us to hypothesize that HUCBCs exerted their effect on reducing cerebral amyloidosis by impacting host immune responses. Although the exact mechanism is still unknown, evidence strongly suggests this therapeutic effect is accomplished by a diminution of Th1 and augmentation of Th2 responses. Importantly, the CD40-CD40L pathway is a key receptor/ligand dyad in initiating and promoting the adaptive immune Th1 response and innate immune responses in mononuclear cells. CD40 receptor, a member of the TNF and nerve growth factor receptor super-family, is expressed on many professional and non-professional APCs, including dendritic cells, B cells, monocytes/macrophages and microglial cells [184-188]. Furthermore, soluble CD40L was shown to be elevated in MS and AD patients [189,190]. Based on the conspicuous role of the CD40-CD40L interaction in mediating brain pro-inflammatory responses and exacerbating AD-like pathology, we investigated the impact of HUCBC administration to AD mice on CD40 pathway-mediated immune responses [186]. Our results show decreased expression of microglial CD40 and reduction in both CNS and peripheral sCD40L concomitant with HUCBC-induced diminished AD-like pathology, raising the possibility that disruption of CD40-CD40L interaction may be responsible for mitigation of AD-like pathology in this scenario [183]. These results are in accord with those of Vendrame and colleagues, who observed that HUCBC administration conferred rescue of behavioral impairment and brain pathology in mouse models of stroke via upregulation of Th2, and downregulation of Th1 cytokine responses [178].

## Flavonoids: a natural strategy to modulate immune responses in AD

A third means by which the aberrant pro-inflammatory responses of inflammaging may be countered thereby reducing AD severity is a group of natural plant-derived compounds known as polyphenols; specifically those known as "flavonoids" from the green tea plant. We and others have focused on the treatment potential of a group of flavonoids, which have been demonstrated to be both anti-inflammatory and anti-amyloidogenic in the context of AD [191-195]. Flavonoids are a large family of compounds synthesized by plants that have a common chemical structure [196]. Extensive analysis of the polyphenolic components of green tea have been conducted. Green tea flavonoids like epigallocatechin gallate (EGCG) appear to promote downregulation of innate immune cell functions. Putative mechanisms of flavonoid action on the innate immune system include direct free radical scavenging [197,198] as well as reduction of inflammatory cytokine production including TNF-α, IL-1β, and prostaglandin E2 in response to LPS-induced activation [199]. In accord with these findings, activated microglia co-cultured with neuroblastoma cells were less neurotoxic in the presence of the flavonoid fisetin, suggesting that some flavonoids may act to inhibit proinflammatory innate immune responses [200,201]. Some flavonoids, including silibinin and EGCG, may modulate adaptive T-cell mediated immune function by downregulating innate immune stimulating cytokines that promote Th1 immunity (e.g., TNF-α) and by promoting Th2 cytokines. These effects are thought to be mediated in part *via *downregulation of NFκB signaling [202-206]. Recently, we have focused on three flavonoids as having therapeutic potential for AD: EGCG, luteolin, and diosmin.

EGCG inhibits TNF-α-induced production of monocyte chemotactic protein-1 from vascular endothelial cells [207]. Furthermore, EGCG also displays the ability to suppress neuron death mediated by activated microglia [208]. Interestingly, green tea polyphenols and unripened apple polyphenols appear to downregulate CD11b on bovine peripheral monocytes, suggesting that leukocyte adhesion and migration mediated in part by CD11b/CD18 could be inhibited after treatment with these compounds. In the same study, unripened apple polyphenols upregulated CD11b on γδ T cells, indicating that these compounds also act to modulate the adaptive immune response [209]. In addition, EGCG suppresses endocytotic activity, and inhibits stimulatory activity toward allogeneic T cells [166].

Most recently, we found that treatment of AD model neurons (murine N2a cells transfected with the human "Swedish" mutant form of APP) and primary neuronal cells derived from AD model mice (the Tg2576 mouse model of AD [211] with luteolin yielded significant reduction in Aβ generation [212]. This flavonoid achieves this effect *via *selective inactivation of glycogen synthase kinase 3-alpha (GSK-3α). Additionally, we observed that administration of a structurally similar flavonoid, diosmin, to Tg2576 mice similarly reduced Aβ generation, and this effect seems to also occur through GSK-3 inhibition. Luteolin has been previously been shown to be a potent free radical scavenger [213,214], anti-inflammatory agent [215,216], and immunomodulator [217,218], and it is possible that these latter two properties are owed to inhibition of GSK-3. Luteolin is also associated with inhibition of NF-κB nuclear translocation, and p38 MAPK signaling [219-221].

Although flavonoid-rich diets and flavonoid administration prevent cognitive impairment associated with inflammaging in animal studies [222-225], retrospective cohort studies are inconsistent in showing an inverse association between dietary flavonoid (e.g., green tea) intake and dementia or neurodegenerative disease risk in humans [226-229]. For example, an epidemiological study of Dutch adults found total dietary flavonoid intake was not associated with the risk of developing AD [226,227], except in current smokers whose risk of AD decreased by half for every 12 mg increase in daily flavonoid intake. On the other hand, elderly French men and women with the lowest flavonoid intakes had a risk of developing dementia over the next 5 years that was 50% higher than those with the highest intakes [228]. Thus, future human studies (ideally randomized clinical trials) will be required which involve supplementation with relatively high doses of specific purified flavanoids to shed light on the apparent inverse risk relationship with AD (and whether this occurs by reducing inflammaging) and to determine if such compounds are therapeutically beneficial.

## Conclusion

In this review we have attempted to give a perspective on the immunologic aspects of aging as they may relate to AD, particularly the increased innate immunity by cells of the mononuclear phagocyte lineage. While the over-active innate immunity characteristic of inflammaging may remain sub-clinical in many elderly individuals, in others it may represent a significant risk for development of one or more aging-associated diseases, including AD. Bringing all this together, it is clear that the association between inflammation, aging, and AD, as suggested by the wealth of epidemiological, clinical and laboratory data, is based on a series of complex molecular and cellular changes that we are only just beginning to uncover and understand. The initial signs are promising but further work is needed in this area to research dietary, cell-based, and pharmacological strategies which oppose the features of inflammaging and thus allow for prevention or treatment of AD.

## Abbreviations

AD: Alzheimer's disease; IL: Interleukin; TNF: Tumor necrosis factor; EGCG: Epigallocatchin gallate; HUCBC: Human umbilical cord blood cells.

## Competing interests

The authors declare that they have no competing interests.

## Authors' contributions

The authors made the following contributions. BG did the majority of the writing and compilation of the literature. DO wrote the Abeta vaccination section. AT provided editing of the entire manuscript as did EO. FF provided clinical consultion. TT edited the entire manuscript and provided consultation on microglial activation and flavonoids. Finally, JT wrote the section on HUCBC and also edited the entire manuscript.
